# Delayed presentation of aortoenteric fistula after esophagectomy

**DOI:** 10.1093/jscr/rjaf674

**Published:** 2025-08-29

**Authors:** Maya Shah, Peter H Liu, Nicholas Iglesias, Raphael Lee, Alan S Livingstone

**Affiliations:** University of Miami Miller School of Medicine, 1600 NW 10th Ave, Miami, FL 33136, United States; Department of Surgery, University of Miami Miller School of Medicine, 1475 NW 12th Ave, Miami, FL 33136, United States; Division of Surgical Oncology at Department of Surgery, University of Miami Miller School of Medicine, Sylvester Comprehensive Cancer Center, 1475 NW 12th Ave, Miami, FL 33136, United States; Department of Surgery, University of Miami Miller School of Medicine, 1475 NW 12th Ave, Miami, FL 33136, United States; University of Miami Miller School of Medicine, 1600 NW 10th Ave, Miami, FL 33136, United States; Department of Surgery, University of Miami Miller School of Medicine, 1475 NW 12th Ave, Miami, FL 33136, United States; Division of Surgical Oncology at Department of Surgery, University of Miami Miller School of Medicine, Sylvester Comprehensive Cancer Center, 1475 NW 12th Ave, Miami, FL 33136, United States

**Keywords:** aortoenteric fistula, esophagectomy, gastric conduit, thoracic aorta, delayed complication

## Abstract

Aortoenteric fistula (AEF) is a rare but catastrophic complication following esophagectomy, often resulting in massive upper gastrointestinal bleeding and high mortality. While most cases occur early in the postoperative period, delayed presentations remain poorly understood. We report the case of a 54-year-old woman who underwent esophagectomy and gastric pull-up for mid-esophageal squamous cell carcinoma. Nine months postoperatively, the patient presented with hematemesis, hemorrhagic shock, and died despite resuscitative efforts. Autopsy revealed a 0.9 cm erosion in the descending thoracic aorta with adjacent ulceration of the gastric conduit and bacterial infiltration consistent with a thoracic AEF. This case illustrates a rare, delayed presentation of AEF confirmed by post-mortem analysis. It highlights the need for ongoing clinical vigilance even in patients with initially uncomplicated recoveries.

## Introduction

An aortoenteric fistula (AEF) is a rare, life-threatening complication following esophagectomy, causing massive upper gastrointestinal bleeding [1, 2]. Gastric conduits brought through the posterior mediastinum during reconstruction are positioned in close proximity to the aorta, trachea, and bronchi. The development of a fistula from the conduit to the aorta is an aortogastric fistula, a subtype of AEF. AEFs are typically attributed to early postoperative complications like infection, anastomotic leak, or radiation injury, and commonly present within weeks of surgery [3]. Delayed AEFs occurring months or even years later have been reported, though they remain exceedingly rare and often escape early detection [4].

We report a rare case of a delayed thoracic AEF nine months after esophagectomy for mid-esophageal squamous cell carcinoma (SCC). Tumor adherence to the descending thoracic aorta required conversion from a transhiatal approach to a McKeown thoracotomy. Despite an initially uneventful postoperative recovery, she presented nine months later with catastrophic upper gastrointestinal bleeding and was found post-mortem to have gastric ulceration fistulizing to the aorta.

## Case report

**Figure 1:**
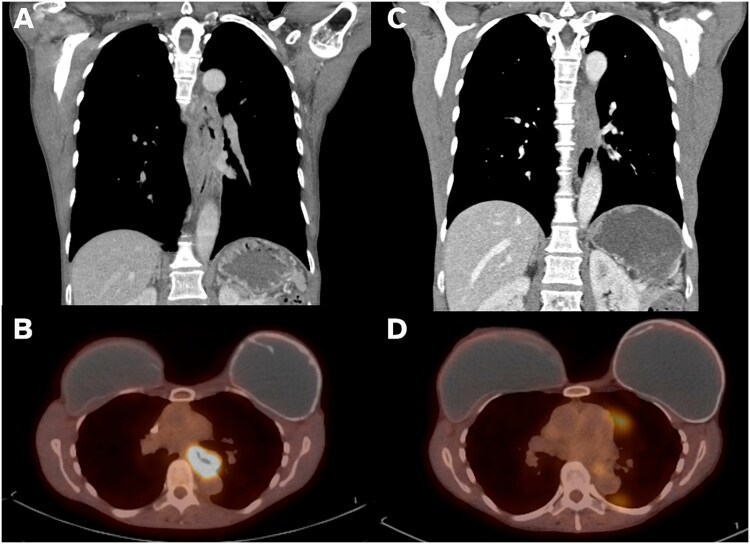
Imaging findings on presentation vs post-neoadjuvant therapy. (A) Coronal CT imaging showing 8.6-cm long esophageal mass. (B) PET imaging with mid-esophageal mass with SUV 15.4. (C) Post-CROSS protocol esophageal mass with significantly improved wall thickening on coronal CT. (D) Post-CROSS PET with decreased FDG avidity at the level of the mid esophagus.

A 54-year-old non-smoking female presented with food impaction and progressive dysphagia. Workup revealed a T3N1M0 circumferential keratinizing invasive SCC of the mid-esophagus extending from 27-cm to 36-cm from the incisors. Pathologic-appearing lymph nodes were noted on endoscopic ultrasound. Computed tomography (CT) imaging reported an 8.6-cm long esophageal mass starting above the carina and adjacent but not involving the thoracic aorta (Fig. 1A). The mass was FDG avid (SUV 15.4) without distant metastases (Fig. 1B). Immunohistochemistry showed intact mismatch repair proteins, microsatellite stability, and low PDL1 expression. The patient received neoadjuvant CROSS protocol (carboplatin/paclitaxel and 23 fractions of 41.4 Gy of radiation). Repeat imaging showed significantly improved wall thickening (Fig. 1C) and decreased FDG avidity (SUV 3.2, Fig. 1D). Endoscopy showed a 10-mm mid-esophageal stricture with repeat negative biopsies. The planned transhiatal esophagectomy was converted to a right thoracotomy intraoperatively due to the esophagus being densely adherent at the carina and descending thoracic aorta. The esophagectomy was completed trans-thoracically avoiding injury to the trachea or aorta and securing clear margins. The stomach was brought up through the posterior mediastinum and anastomosed in the neck to the cervical esophagus. The patient recovered uneventfully and was discharged on postoperative day 10.

Pathology revealed a partial response to neoadjuvant therapy (grade 2) and no residual tumor on the descending thoracic aorta, trachea, or mainstem bronchi. The tumor had no lymphovascular invasion or perineural invasion. Of 18 lymph nodes, 2 lymph nodes were positive for metastatic disease. Final staging was a ypT2N1M0 SCC of the middle third of the esophagus.

Postoperatively, rising circulating tumor DNA (ctDNA) level was noted despite the absence of radiographic evidence of disease. She was started on pembrolizumab, carboplatin, and paclitaxel. When ctDNA continued to rise, she was escalated to fluorouracil, leucovorin, oxaliplatin and docetaxel in combination with pembrolizumab and completed six cycles. A lower posterior mediastinal lymph node was biopsy proven to have recurrence and was treated with 35 Gy of radiation in five fractions.

Nine months following surgery, she presented with massive hematemesis and shock. Massive transfusion protocol was implemented, but she rapidly deteriorated and had a cardiac arrest before she could undergo endoscopy or CT angiography (CTA). Despite aggressive resuscitation, she quickly expired.

**Figure 2:**
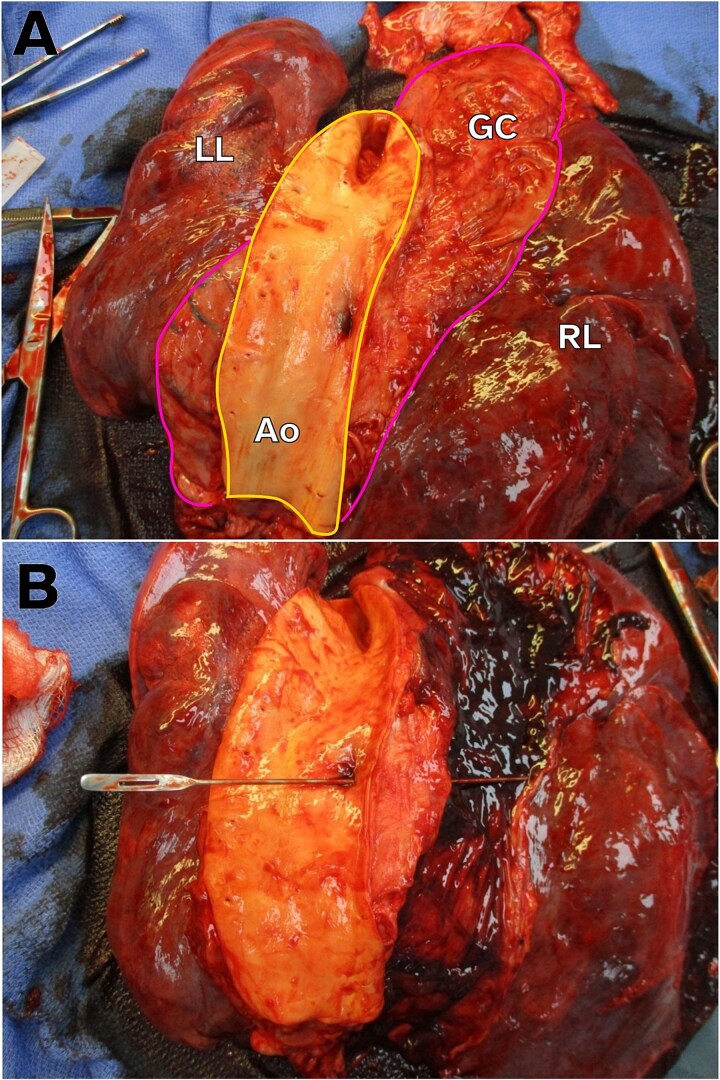
Gross autopsy images showing AEF. (A) Image of the intra-thoracic structures from the posterior-to-anterior view with the aorta opened. For orientation, the aorta is outlined in yellow and the gastric conduit outlined in pink. Note the ulceration on the aorta side of the AEF. LL, left lung. RL, right lung. GC, gastric conduit. Ao, aorta. (B) Probe traversing the AEF from the aorta into the gastric conduit, which is now opened, containing intra-luminal hemorrhage.

Post-mortem examination revealed a 0.9-cm gastric ulcer with gram-positive cocci penetrating into the descending thoracic aorta (Fig. 2A and B) producing an AEF. Minute foci of treated SCC were in the vicinity of the ulcer, but the bacterial infection appeared to be the cause of the fistula.

## Discussion

AEF following esophagectomy is a rare complication with high mortality, often occurring within weeks of surgery from acute infection, anastomotic leak, or conduit ischemia [3, 4]. Our patient developed a thoracic AEF nine months postoperatively, without preceding leak, infection, or sentinel bleed, highlighting the potential for delayed and silent progression. Autopsy revealed a gastric conduit ulcer with transmural bacterial infiltration eroding into the descending thoracic aorta, suggesting a chronic and indolent process culminating in sudden fatal hemorrhage.

Delayed thoracic AEFs are exceedingly uncommon, with only a handful of documented cases and even fewer substantiated by autopsy. Nishimura et al. described an AEF 11 years post-esophagectomy, attributed to chronic mechanical irritation from a staple line [5]; other cases have postulated radiation-induced vasculopathy and persistent inflammation as potential contributors [1, 6]. Our case is characterized by the convergence of several high-risk features: tumor adherence to the aorta, complex dissection, chemoradiation, and subsequent subclinical gram-positive conduit ulceration.

This case invites careful reflection on surveillance strategies in post-esophagectomy patients with known vascular proximity. Current guidelines do not advocate routine vascular imaging in asymptomatic individuals; however, in patients with known aortic involvement, consideration may be given to tailored surveillance protocols employing periodic contrast-enhanced CT or endoscopy to detect early mucosal compromise or impending conduit-aortic erosion [7, 8]. Notably, our patient remained clinically stable until the final presentation with no warning signs on routine postoperative imaging that might have prompted a preemptive intervention.

In exsanguinating patients, resuscitative endovascular balloon occlusion of the aorta (REBOA) represents a potentially life-saving temporizing adjunct. It does not require CTA and placement of a Zone 1 REBOA catheter has demonstrated utility in trauma and selected cases of non-variceal gastrointestinal hemorrhage [9, 10]. While REBOA would not have addressed the underlying pathology in this case, it might have facilitated transient hemodynamic stabilization for definitive surgical or endovascular control. Unfortunately, the fulminant nature of our patient’s presentation precluded even this temporizing strategy. Thoracic endovascular aortic repair (TEVAR) also offers rapid exclusion of the fistula and hemorrhage control in AEF [11]. Although not always curative without concomitant management of the infected conduit, TEVAR might serve as a bridge to surgical reconstruction in selected patients.

This case underscores the need for long-term vigilance in post-esophagectomy patients, especially when the conduit abuts major vascular structures. Delayed AEF may arise without warning signs and may present suddenly and fatally. Clinicians should maintain a high index of suspicion in any post-esophagectomy patient with unexplained upper gastrointestinal bleeding or hemodynamic instability.
